# Dosimetric study of three‐dimensional static and dynamic SBRT radiotherapy for hepatocellular carcinoma based on 4DCT image deformable registration

**DOI:** 10.1002/acm2.12811

**Published:** 2019-12-30

**Authors:** Changdong Ma, Jinghao Duan, Shuang Yu, Changsheng Ma

**Affiliations:** ^1^ Department of Radiation Therapy Qilu Hospital of Shandong University Jinan 250012 China; ^2^ Department of Radiotherapy Shandong Cancer Hospital and Institute Shandong First Medical University and Shandong Academy of Medical Sciences Jinan Shandong Province 250117 China

**Keywords:** 4D radiotherapy, deformable image registration, four‐dimensional (4D) computed tomography, hepatocellular carcinoma

## Abstract

The purpose of this work was to determine the actual dose received by normal tissues during four‐dimensional radiation therapy (4DRT) composed of ten phases of four‐dimensional computer tomography (4DCT) images. The analysis was performed by tracking the hepatocellular carcinoma SBRT. Data were acquired from the tracking of each phase with the beam aperture for 28 hepatocellular carcinoma patients, and the data were used to generate a cumulative plan, which was compared to a three‐dimensional (3D) plan formed from a merged target volume based on 4DCT images in a radiation treatment planning system (TPS). The change in normal tissue dose was evaluated in the plan using the parameters V5, V10, V15, V20, V25, V30, V35, and V40 (volumes receiving 5, 10, 15, 20, 25, 30, 35, and 40 Gy, respectively) in the dose‐volume histogram for the liver; the mean dose was analyzed for the following tissues: liver, left kidney, and right kidney. The maximum dose was analyzed for the following tissues: bowel, duodenum, esophagus, stomach, and heart. There was a significant difference in the dose between the 4D planning target volume (PTV) (average 115.71 cm^3^) and ITV (169.86 cm^3^). The planning objective was for 95% of the volume of the PTV to be covered by the prescription dose, but the mean dose for the liver, left kidney and right kidney had an average decrease of 23.13%, 49.51%, and 54.38%, respectively. The maximum dose for the bowel, duodenum, esophagus, stomach, and heart had an average decrease of 16.77%, 28.07%, 24.28%, 4.89%, and 4.45%, respectively. Compared to 3D RT, the radiation volume for the liver V5, V10, V15, V20, V25, V30, V35, and V40 using the 4D plans had a significant decrease (*P *﹤ 0.05). The 4D method creates plans that permit sparing of the normal tissues more than the commonly used ITV method, which delivers the same dosimetric effects to the target.

## 1 INTRODUCTION

Radiotherapy for inoperable primary and metastatic hepatocellular carcinomas has become feasible with three‐dimensional radiation therapy (3DCRT) treatment planning and treatment delivery. Liver radiotherapy remains challenging because of respiratory motion.^1^ Four‐dimensional computed tomography (4DCT), developed for radiotherapy treatment planning, is a dynamic volume imaging system for moving organs with an image quality comparable to that of conventional CT.^2^ In recent years, 4DCT techniques have been used in clinical RT practice for hepatocellular carcinoma SBRT and other cancers, and they have shown promising results. The conventional 3D plans can result in geometric misses and include excess normal tissues. Thus, using 4DCT‐based individualized internal target volume (ITV) for hepatocellular carcinoma plans can reduce the target volumes to spare more normal tissues and allow less off‐target dosing compared with 3D plans.^3^, ^4^, ^5^, ^6^

A new technique of target tracking to specifically manage the detrimental effect of respiration on the delivered dose distribution has arisen in recent years. In 4D radiotherapy, the treatment plan is designed on each 4D CT image set (i.e., 4D treatment planning), and radiation is delivered throughout the patient’s breathing cycle (i.e., 4D treatment delivery), which ensures adequate coverage of the tumor target without increasing the treated volume. However, the distinction in possible benefits between the two strategies may not be clear. Based on the assumption that each strategy is designed to achieve target coverage, it would be useful to know the relative difference in normal tissue dose provided by the two strategies. For a given patient, we want to determine the difference in liver dose among the strategies, assuming the delivery with the motion management strategy in question is ideal and without error. To achieve this aim, we evaluated the cumulative target tracking dose plans and 4DCT‐based individualized ITV for hepatocellular carcinoma methods. Radiation‐induced liver disease (RILD) is one of the most important treatment‐related complications in the reports of hepatic irradiation. Dosimetric analysis has shown a correlation between dose‐volume parameters and the risk of RILD. The purpose of this study was to define the potential impact of dosimetric differences in 3D and 4D planning for patients with hepatocellular carcinoma and to estimate the normal tissue complication probability (NTCP) of RILD.

## 2 MATERIALS AND METHODS

### 2.1 Patients

We included 28 patients with pathologically proven HCC. These patients were randomly selected from a list of patients who were treated in our hospital between March 2010 and January 2018. There were 12 women and 16 men with an average age of 56 years (age range: 52–60 years).

### 2.2 4DCT simulation and planning target volume (PTV) acquisition

Simulations were performed with a Philips Brilliance CT Big Bore (Phillips Medical Systems, 96 Highland Heights, OH, USA) connected to a Varian Real‐Time Position Management system (Varian Medical Systems, Palo Alto, CA). The patients were immobilized with a vacuum pillow with their hands above their head. The CT scanning region extended from 4 cm above the upper edge of the diaphragm to 4 cm below the lower edge of the right kidney, with a 3‐mm reconstruction slice thickness. These 4D CT data sets were comprised of a total of 28 CT scans per patient, taken at equally spaced intervals across the entire respiratory cycle (phase‐based sorting in 4D CT reconstruction)^7^.The 4DCT images were transmitted to the treatment planning system (TPS) Varian Eclipse V8.6.15 (Varian Medical Systems, Palo Alto, CA, USA) for target volume contouring and treatment method designing.

GTVs were contoured under the same window width (200 Hu) and level (40 Hu) on each phase of the 4DCT images. PTVs were obtained using 5 mm margins for setup errors. The liver, left kidney, right kidney, bowel, duodenum, esophagus, stomach, and heart were also delineated. A normal liver was defined as the volume of the liver minus the PTV.

### 2.3 Radiotherapy plan design

1. 3D plan design: ① 3D plan: Five fields were used in the 3D CRT plan designing protocol based on the 4DCT image in the merged ITV. ② The prescribed dose of PTV is 5.0 Gy × 10 fractions. We normalized the plan so that the 95% volume of PTV achieved the prescribed dose in the Eclipse 13.6 vision TPS. ③ The dose constraints for OAR were as follows: the mean dose to the normal liver was limited to 23 Gy, and the dose–volume histogram (DVH) of the normal liver was within the tolerance area (i.e., V5 < 86%, V10 < 68%, V20 < 49%, V30 < 28%, and V40 < 20%)^8^; for the stomach and duodenum, the maximum dose was limited to 45 Gy, and the volume receiving> 25 Gy was limited to < 5 cm^3^.^9^
2. 4D plan design: ① Each 4D phase plan was designed with the same field angles as those used in the 3D plan. The 4D dose was obtained by summing the mapped doses from individual phases of the 4D CT using deformable image registration (DIR). ② For each phase plan the set prescribed dose is 0.5 Gy, and the 95% volume of PTV is covered by the prescribed dose. ③ The 4D plan used all phase plans (CT0, CT10,…, and CT90), which were added by the DIR function in MIM Maestro 6.6.9(MIM) (MIM Software Inc., America). ④The 4DCT images, RT structures and RT dose were imported into the MIM Maestro workstation. In this study, the rigid registration was defined automatically using the whole body as a starting point for the region of interest (ROI) for deformation. Deformable registration was performed using the intensity‐based free‐form transformation (FFD) algorithm.^23^, ^24^

### 2.4 Plan evaluation

The 4D dose distribution was compared with the 3D dose of the ITV method. The 3D plan prescription dose was 50 Gy. The fraction dose was 5 Gy. The planning objective was 95% volume of PTV covered by the prescription dose. The liver V5, V10, V15, V20, V25, V30, V35, and V40; mean dose for the liver; and NTCP values were also calculated for each OAR with the Lyman‐Kutcher‐Burman (LKB) model. Three clinical endpoints of liver were considered: change in ALBI, change in Child‐Pugh (C‐P) score and grade 3 or higher liver enzymatic changes. The endpoint of kidney is nephritis. Three clinical endpoints of bowel and duodenum were considered: obstruction, perforation, and stenosis. The endpoint of esophagus is clinical structure and esophagitis, grade≧ 2.The endpoint of heart is pericarditis of any grade. The endpoint of stomach is ulceration or perforation. All the organs were studied in RTOG/ EORTC acute and late radiation injury grading standard. The three parameters were derived according to Burman identification; several parameters, including mean hepatic dose, percent volume of normal liver with radiation dose more than 30 Gy (V30 Gy), and NTCP were calculated from the DVH. The NTCP model of Lyman was used.^22^, ^26^ In the NTCP model.

$$\begin{array}{c} NTCP=1/\sqrt{2\pi }\int_{-\infty }^{t}\exp(-t^{2}/2)dt\\ t=\left(D-TD_{50}\left(v\right)\right)/\left(m\times TD_{50}\left(v\right)\right) \end{array}$$

$$v=V/V_{\mathrm{ref}}$$

TD50(v) was the 50% tolerance dose for uniform irradiation of the partial volume v. The partial and whole liver radiation tolerance doses were related by a power law relationship:

$$\text{TD}\;\left(1\right)=\text{TD}\;\left(v\right)\times v^{n}$$

V_ref_ was the volume of normal liver. The parameter “n” was the volume effect parameter, and the value of 0.32 from the literature was applied. The parameter “m” was the steepness of the dose‐complication curve for a fixed partial volume, and the estimate of 0.15 was used. TD_50_ of 40 Gy was applied in the calculation. The effective volume method of Kutcher and Burman was used to provide estimates of equivalent dose and volume pairs for uniform partial organ irradiation from the DVHs summarizing the nonuniform irradiation.

The dose for the left and right kidney and the maximum dose for the bowel, duodenum, esophagus, stomach, and heart were evaluated. Evaluation parameters for the 4D plan were the same as the 3D plan.

#### 2.4.1 Statistical analysis

**Figure 1:**
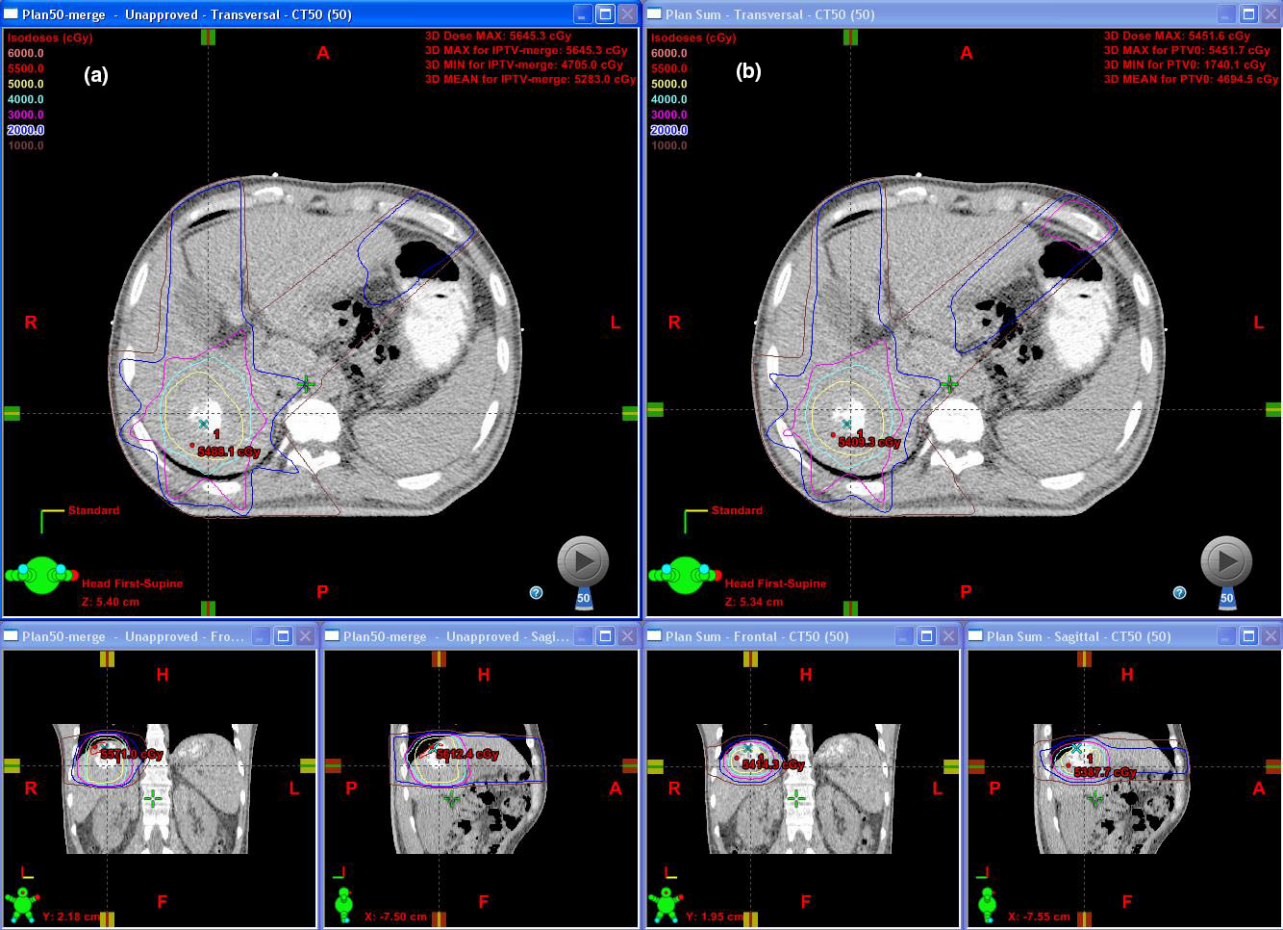
An example of the dose distribution, (a) three‐dimensional (3D) dose and (b) four‐dimensional (4D) dose.

**Figure 2:**
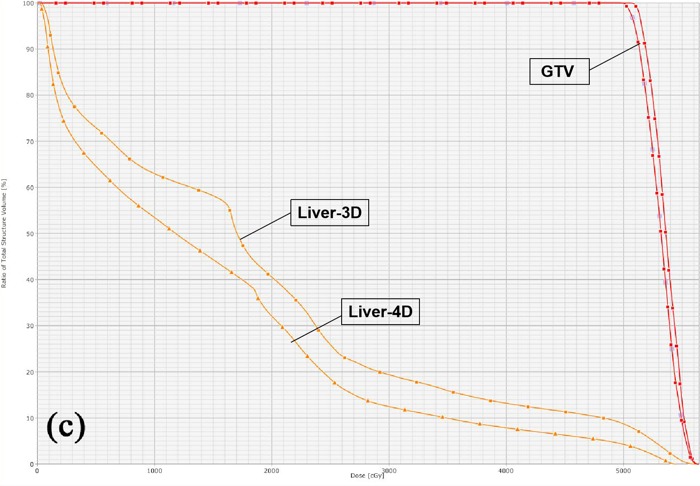
(c) The average dose–volume histogram (DVH) of the 28 patients from the three‐dimensional dose with rectangle symbols and the four‐dimensional dose with triangle symbols. For DVH red line represent GTV and pink line represent Liver.

**Figure 3:**
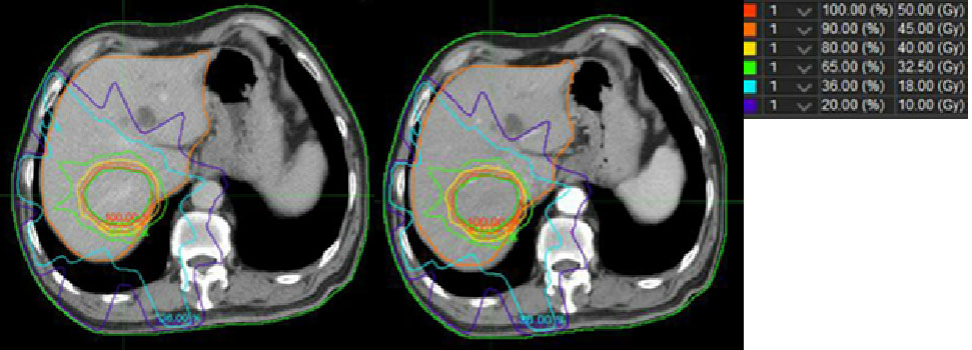
An example of the dose distribution for Liver dose accumulation and plan dose.

The Statistical Package for Social Sciences, version 19.0 (SPSS 19.0) was used for statistical analysis. The Wilcoxon matched pairs signed rank test was performed. Two‐tailed *P* < 0.05 was defined as having statistical significance Figs. 1, 2, 3.

## 3 RESULTS

### 3.1 Target volume comparison

For the 28 cases, the average volume of each phase of the 4DCT image for PTV was 115.71 cm^3^, while the average merged PTV volume was 169.86 cm^3^. There was a significant difference between them.

### 3.2 Dose evaluation

While the planning objective was 95% volume of PTV covered by the prescription dose, the mean dose for the liver, left kidney and right kidney had an average decrease of 23.13%, 49.51%, and 54.38%, respectively. The maximum dose for the bowel, duodenum, esophagus, stomach and heart had an average decrease of 16.77%, 28.07%, 24.28%, 4.89%, and 4.45%, respectively. Table 1 shows details about 28 patients used in the study such as the target volume, the maximum motion etc. Table 2 shows dosimetric changes of OARs for ten cases using the 3D and 4D plans. Table 3 shows the radiation volume for the liver V5, V10, V15, V20, V25, V30, V35, and V40 when using the 3D and the 4D plans (P ﹤ 0.05).

**Table 1:** General clinical data of patients.

Characteristics	Value
Gender	
Male	16
Female	12
Age	52 − 60 y, median 56
4D‐PTV/cm3	115.71 ± 8.1 cm^3^ Range (88.69–136.58)
3D‐PTV/cm3	169.86 ± 30.27 cm3 Range (144.65–217.43)
Liver/cm3	1177.52 ± 434.65cm^3^ Range (944.65–1597.43)
Center of GTV（X‐distance）/cm	0.30 ± 0.20cm Range (0.10–0.50)
Center of GTV（Y‐distance）/cm	0.80 ± 0.30cm Range (0.50–1.10)
Center of GTV（Z‐distance）/cm	1.20 ± 0.50cm Range (0.80–1.70)
Center of GTV（Total‐distance）/cm	1.50 ± 0.60cm Range (0.90–2.10)

**Table 2:** Dosimetric changes of OARs in D4 plans compared to three‐dimensional (3D) plans.

Dose (Gy)	3D plans	4D plans	*t* value	*P* value
Mean liver dose	1893.34 ± 603.50	1455.26 ± 575.12	−10.671	0.000
Mean left kidney dose	103.26 ± 72.22	52.46 ± 35.45	−2.780	0.050
Mean right kidney dose	228.12 ± 307.78	104.48 ± 94.97	−0.915	0.412
Max bowel dose	1401.94 ± 1516.53	1166.42 ± 1374.23	−3.009	0.040
Max duodenum dose	2280.44 ± 1259.80	1640.70 ± 1282.92	−2.038	0.111
Max esophagus dose	1017.36 ± 869.21	770.20 ± 666.12	−1.382	0.239
Max stomach dose	2043.86 ± 1388.74	1943.64 ± 1332.57	−1.096	0.335
Max heart dose	1976.28 ± 2180.73	1888.86 ± 2104.21	−0.444	0.680
NTCP of liver (%)	6.25 ± 2.12	3.05 ± 1.87	−2.786	0.001
NTCP of left kidney (%)	0	0	No	No
NTCP of right kidney(%)	0	0	No	No
NTCP of bowel (%)	2.05 ± 1.80	1.09 ± 1.04	−1.076	0.080
NTCP of duodenum (%)	2.02 ± 0.89	0.82 ± 0.612	−2.967	0.049
NTCP of esophagus (%)	1.46 ± 0.98	0.80 ± 0.56	−3.098	0.046
NTCP of stomach (%)	1.12 ± 0.59	1.01 ± 0.75	−1.764	0.087
NTCP of heart (%)	5.04 ± 1.92	4.61 ± 1.68	−1.897	0.083

Note

Statistically significant differences (*P* < 0.05).

**Table 3:** Radiation volume for lung V5, V10, V15, V30, and V50 using the three‐dimensional (3D) plans and the four‐dimensional (4D) plans.

Radiation volume (%)	3D plans	4D plans	*t* value	*P* value
Liver V5	63.85 ± 16.43	53.36 ± 17.43	−7.986	0.001
Liver V10	55.07 ± 16.62	44.55 ± 16.53	−9.594	0.001
Liver V15	50.74 ± 16.30	39.08 ± 16.18	−10.329	0.000
Liver V20	42.41 ± 15.52	30.87 ± 15.57	−9.812	0.001
Liver V25	28.47 ± 12.24	20.20 ± 10.62	−8.160	0.001
Liver V30	23.58 ± 11.76	16.53 ± 9.83	−7.564	0.002
Liver V35	20.41 ± 11.18	14.28 ± 9.33	−6.787	0.002
Liver V40	17.44 ± 10.29	12.13 ± 8.66	−6.626	0.003

Note

Statistically significant differences (*P* < 0.05).

## 4 DISCUSSION

Our findings suggest that the 4D planning method is an effective means of treatment; it has features that make it superior to the 3D ITV method, which currently is the most common strategy implemented clinically to compensate for respiration‐induced target motion. Essentially, the 4D plan method uses a smaller PTV, while using a similar target dose distribution of the planning CT. Because the 4D planning method accounts for the effects of respiratory motion by adjusting the dose within the target, the margin can be reduced relative to that in the ITV method plan, leading to less off‐target dosing of normal tissues.^10^

Most centers have the ability to acquire 4D CT images, but they do not have the ability to perform 4D radiation delivery. Instead, 4D CT images are primarily used to define the ITV, which is essentially the envelope needed to enclose the target as it moves throughout the breathing cycle. The major tasks in 4D‐RT are fundamentally the same as those that are currently in practice for 3D‐RT. The workflow involves the key tasks of image acquisition, target delineation, and treatment planning and delivery. However, the process can be significantly more involved in its most explicit implementation.

To estimate a realistic dose delivered to the patients in the presence of respiratory motion, a four‐dimensional dose calculation (4D dose) using DIR of 4DCT images has been studied.^11^, ^12^, ^13^, ^14^, ^15^, ^16^ In this study, we performed a 4D dose calculation. The 4D dose delivered to the target volume and normal organs during free‐breathing RT for hepatocellular carcinoma was calculated using hybrid DIR for all phase images from 4DCT with the Finite Element Model. The goal was to evaluate the relative difference in the liver dose between an ideal implementation of the strategy and a 3D plan dose based on ITV of 4DCT. Differences were found between the 4D target tracking dose and the 3D dose. Based on these results, we can conclude that this difference was due to the movement itself. As expected, the strongest factor of producing a relative difference in liver dose was the amplitude of tumor excursion into respiration. The target tracking dose is delivered throughout the breathing cycle. The larger volume of the liver at the end of expiration has been shown to reduce the dose to the liver for a given beam aperture.^11^, ^17^, ^18^, ^19^, ^20^, ^21^, ^22^

In MIM Maestro, a rigid registration is initially applied, which is followed by a nonrigid registration. In this study, the rigid registration was defined automatically using the whole body as a starting point of the ROI for deformation. Nonrigid registration was performed using the intensity‐based FFD algorithm. If the respiratory tumor motion is large, the DIR system needs to perform a large deformation to match the two images. However, in the current study, the magnitude of the respiratory motion is low. So, the accuracy of deform performed using our specifically 4DCT study.^23^, ^24^ The process of dose accumulation in this study was as follows: First, the corresponding dose distribution was obtained by designing the plan on 0% phase of 4DCT images, and then the dose was deformed to all phases of 4DCT images to acquire the dose on all phases, finally, the dose of each phase was performed the dose accumulation. All of the above procedures are accomplished by the function of "Deformable Dose Accumulation" provided with the MIM software.

Four‐dimensional image positioning technology has been mature and commercialized. With the development of imaging technology, multi‐leaf grating and mechanical control technology, the real‐time tracking down tumor motion during the treatment of patients can make the beam follow the target tumor in real time, which becomes the development direction of tumor motion compensation. The advantage of real‐time tracking is that the working cycle of the linear accelerator will not lose as much as that of the breathing threshold method, so the treatment time will not be prolonged. The most commonly used tracking method is real‐time imaging tracking down moving tumors based on x‐ray fluoroscopy. There is also electromagnetic‐based tracking technology. During the implementation of the four‐dimensional therapy, besides real‐time tracking of tumors, the implementation of the treatment has response time to changes in respiratory phase. Prediction software is needed to reduce response time errors. With the development of these technologies, the implementation of real four‐dimensional radiotherapy will become possible soon.

The purpose of this study is to evaluate whether the four‐dimensional plan to design process can spare the normal tissues more than the commonly used ITV method of the implementation of 4D therapy, thereby reducing cumulative dose in the implementation of 4D therapy, indirectly improving the therapeutic effect, and improving the operability of clinical application of 4D radiotherapy. This study is based only on dosimetric studies. However, how to achieve four‐dimensional radiotherapy still faces many challenges.

Respiratory‐guided gating (true four‐dimensional radiotherapy): It refers to the 4DRT (four‐dimensional radiotherapy technology) based on the images obtained by 4DCT (four‐dimensional CT). Its working principle is not to control the patient's breathing, only to monitor the patient's breathing, so as to control the scanning of four‐dimensional cone‐beam CT, that is, to take different breathing phases of patients. Collecting the respective respiratory images and then outlining the respective target areas (GTV, CTV, PTV, etc.) in each phase of the image. In radiotherapy, the breath of patients is monitored with different breathing phases using different radiation plans.

RPM gating technology is one of the most popular respiratory gating methods in the world in recent years. Through RPM gating technology, a single breathing phase CT image can be taken for contour mapping and planning design of intensity modulation planning target area and organs at risk. In the course of radiotherapy, the marker points of the patient's body surface are recognized by the infrared camera of RPM system, and the individual respiratory wave is drawn. Only when the respiratory movement reaches the corresponding phase of planned image, the beam/stop therapy can be automatically made. The volume of planned target irradiation can be effectively reduced, and the irradiated dose and toxic side effects of normal tissues can be reduced.

Due to radiotherapy equipment and radiotherapy technology, the current respiratory gated radiotherapy technology can only be developed in a small number of radiotherapy units. Of course, there are still many problems of breath‐gated radiotherapy: how to choose the appropriate time of gating, how to design radiotherapy plan quickly, how to shorten the treatment time, and so on. There are many limitations: selection of gated phase, study on the range of motion of tumors under gating technique, study on PTV volume under gating technology, and so on.^25^, ^26^, ^27^

The free form deformable registration algorithm is used in the registration algorithm between different phases of 4DCT, which has strong robustness to image noise due to its deformation constraints. Respiratory artifacts have a direct impact on registration errors, mainly in the lower part of the lung and the diaphragm. The size of the artifact is mainly determined by the scanning time and scanning mode. We use the cine mode scanning, the average of the head foot direction artifact of the upper diaphragm is 0.5 cm for each scanning circle of 0.3 seconds. No significant respiratory artifacts were found on 4DCT. In addition, the error of dose accumulation is not only related to registration error, but also related to dose flatness. If the tumor is near the diaphragm and the dose gradient at the edge of the tumor is large, the larger registration error in this area will also produce larger dose accumulation error. If the tumor is far away from the diaphragm, the diaphragm is located in the low dose area, and the dose gradient is small, although there is a large registration error in this area, the effect on the cumulative dose is small.

## 5 CONCLUSION

The 4D method is an effective and practical way to design treatment plans for tumors subject to respiratory motion. The 4D planning method has better targeting, which spares the normal tissues more than the commonly used ITV method, all while delivering the same dosimetric effects to the target.

## CONFLICT OF INTEREST

The authors declare no conflict of interest.
